# Interplay Between ROS and Hormones in Plant Defense Against Pathogens

**DOI:** 10.3390/plants14091297

**Published:** 2025-04-25

**Authors:** Mostafa Haghpanah, Amin Namdari, Mostafa Koozehgar Kaleji, Azam Nikbakht-dehkordi, Ahmad Arzani, Fabrizio Araniti

**Affiliations:** 1 Dryland Agricultural Research Institute (DARI), Agriculture Research, Education and Extension Organization (AREEO), Gachsaran 7589172050, Iran; masoudhgh@gmail.com (M.H.); aminnamdari61@gmail.com (A.N.); 2 Department of Agronomy, Faculty of Plant Production, Gorgan University of Agricultural Sciences and Natural Resources, Gorgan 4918943464, Iran; mostafa.koozehgar@gmail.com; 3 Research and Technology Institute of Plant Production, Afzalipour Research Institute, Shahid Bahonar University of Kerman, Kerman 7616914111, Iran; anikbakht@uk.ac.ir; 4 Department of Agronomy and Plant Breeding, College of Agriculture, Isfahan University of Technology, Isfahan 8415683111, Iran; 5 Department of Agricultural and Environmental Sciences—Production, Landscape, Agroenergy, University of Milan, 20133 Milan, MI, Italy

**Keywords:** antioxidants, hypersensitive response, oxidative burst, pathogen recognition, systemic signaling

## Abstract

Reactive oxygen species (ROS) are toxic by-products of aerobic cellular metabolism. However, ROS conduct multiple functions, and specific ROS sources can have beneficial or detrimental effects on plant health. This review explores the complex dynamics of ROS in plant defense mechanisms, focusing on their involvement in basal resistance, hypersensitive response (HR), and systemic acquired resistance (SAR). ROS, including superoxide anion (O^2−^), singlet oxygen (^1^O_2_), hydroxyl radicals (OH), and hydrogen peroxide (H_2_O_2_), are generated through various enzymatic pathways. They may serve to inhibit pathogen growth while also activating defense-related gene expression as signaling molecules. Oxidative damage in cells is mainly attributed to excess ROS production. ROS produce metabolic intermediates that are involved in various signaling pathways. The oxidative burst triggered by pathogen recognition initiates hyper-resistance (HR), a localized programmed cell death restricting pathogen spread. Additionally, ROS facilitate the establishment of SAR by inducing systemic signaling networks that enhance resistance across the plant. The interplay between ROS and phytohormones such as jasmonic acid (JA), salicylic acid (SA), and ethylene (ET) further complicates this regulatory framework, underscoring the importance of ROS in orchestrating both local and systemic defense responses. Grasping these mechanisms is essential for creating strategies that enhance plant resilience to biotic stresses.

## 1. Introduction

In plants, reactive oxygen species (ROS) are constantly generated as byproducts of regular metabolic processes. The types of ROS include Superoxide anion (O_2_^−^) is a highly reactive free radical that can be generated in nearly all subcellular compartments in plants [1]. It is formed as the first product in the chain of ROS production. This process is mainly due to the one-electron reduction of molecular oxygen (O_2_). Hydrogen peroxide (H_2_O_2_) is a non-radical form of ROS. It is relatively stable, capable of crossing cell membranes, and can cause damage to various cellular structures [1,2]. H_2_O_2_ is a signaling molecule involved in multiple distinct physiological processes and stress responses in plants. Among ROS, H_2_O_2_ possesses the longest half-life and can move across cell membranes through aquaporins, enabling it to travel significant distances within the plant [3]. Singlet oxygen (^1^O_2_) is a highly reactive, non-radical ROS and is mainly produced in chloroplasts through energy transfer from excited chlorophyll molecules [4]. Unlike H_2_O_2_, ^1^O_2_ has a very short lifespan. It can cause damage to cellular components. The hydroxyl radical (OH) is highly reactive and can harm many biomolecules. It is typically formed through the Fenton reaction, where hydrogen peroxide interacts with reduced metals like iron or copper [5]. Organic hydroperoxides (ROOH) are a type of ROS formed through oxidation of organic molecules, such as lipids and proteins [6]. Plants also produce other ROS, such as the hydroperoxyl radical (HO_2_) and carbonate radical (CO_3_^−^) [1].

ROS serve a dual role in plant cells, acting as essential signaling molecules at low concentrations [7] while becoming toxic oxidants that can damage cells at high levels [8]. Grasping the significance of ROS in plant physiology and their responses to stress is crucial, especially in plant-pathogen interactions. Plants have enzymatic systems that generate ROS, such as respiratory burst oxidase homologs (OSEs) in the plasma membrane and class III peroxidases in the cell wall [9]. Other ROS-producing enzymes include oxalate oxidases, amine oxidases, lipoxygenases, and quinone reductases [10].

Plants possess sophisticated scavenging systems, including both enzymatic and non-enzymatic antioxidants, to preserve ROS homeostasis [11]. The intricate balance between the production and scavenging of ROS is essential for maintaining the transient and specific nature of ROS signals. This balance allows ROS to effectively modulate various biological processes [12]. Enzymatic antioxidants are proteins that catalyze the breakdown of ROS into less reactive molecules. Plants contain several crucial enzymatic antioxidants [11,13], including (i) superoxide dismutase (SOD), which catalyzes the dismutation of O_2_^−^ into H_2_O_2_ and O_2_, (ii) catalase (CAT), which primarily detoxifies H_2_O_2_ by breaking it down into water and oxygen; (iii) ascorbate peroxidase (APX), which utilizes ascorbate to reduce H_2_O_2_ to water, resulting in the production of dehydroascorbate; and (iv) glutathione peroxidase (GPX), which catalyzes the reduction of H_2_O_2_ and organic hydroperoxides, using glutathione as a reductant. Monodehydroascorbate reductase and dehydroascorbate reductase are involved in regenerating ascorbate, which is essential for APX activity. Glutathione reductase (GR) regenerates glutathione, required for GPX activity [14].

Non-enzymatic antioxidants are small molecules that directly scavenge ROS. For example, glutathione is a tripeptide that functions as a redox buffer and can directly scavenge ROS [1]. Carotenoids are essential for protecting the photosynthetic apparatus from singlet oxygen damage. Tocopherols, lipid-soluble antioxidants, protect cell membranes against lipid peroxidation induced by ROS. Phenolic compounds, including flavonoids and tannins, are a diverse group of polyphenols known for their antioxidant activity and ROS-scavenging properties. Soluble sugars, such as disaccharides and oligosaccharides, can influence the production and scavenging of ROS, potentially regulating ROS-producing pathways or contributing to NADPH production for antioxidant processes [1].

ROS play a crucial role in numerous physiological processes in plants, including growth and development, as well as responses to both biotic and abiotic stresses [15]. ROS interact with plant hormones, such as salicylic acid (SA), jasmonic acid (JA), and ethylene (ET), which play crucial roles in activating induced defense mechanisms in plants [16,17]. Despite the extensive literature on ROS in plants, which focuses either on their destructive cellular role or their function as signaling molecules, a significant gap remains in understanding the dynamics of ROS in plants under pathogen stress. Specifically, there is a lack of comprehensive discussions on how ROS interact with phytohormones to modulate plant defense mechanisms against pathogens. This oversight limits our understanding of the complex interplay between ROS signaling and phytohormone regulation in plant immunity. This review aims to explore the essential role of ROS in plant defense mechanisms, focusing on their contributions to both constitutive and induced resistance. Specifically, this review will delve into the diverse roles of ROS in basal resistance, the hypersensitive response, and systemic acquired resistance, highlighting their interactions with phytohormones to provide a comprehensive understanding of how these components collaborate to enhance plant immunity against pathogens.

### 1.1. Basal Defense

**Figure 1:**
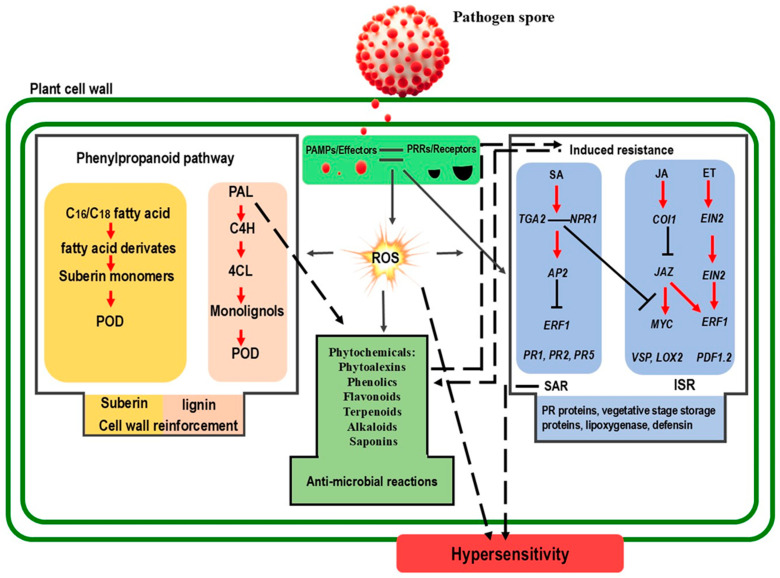
Schematic representation of the interplay between reactive oxygen species (ROS), hormones, and phytochemicals in plant cells during plant-pathogen interactions. The plant cell wall serves as the first line of defense. Pathogen recognition involves the detection of pathogen-associated molecular patterns (PAMPs) and effectors by pattern recognition receptors (PRRs), triggering a cascade of signaling events. These events stimulate various plant hormones, including salicylic acid (SA), jasmonic acid (JA), and ethylene (ET), and induce a burst of ROS. ROS molecules also trigger the production of antimicrobial phytochemicals, activate pathogenesis-related (PR) proteins, and promote the synthesis of lignin and suberin. The involvement of ROS in signaling-induced resistance and local responses, such as hypersensitivity (HR), is also depicted. Key enzymes involved in these processes include phenylalanine ammonia-lyase (PAL), cinnamate 4-hydroxylase (C4H), and 4-coumarate: Coenzyme A ligase (4CL), which are crucial for lignin biosynthesis. Peroxidase (POD) enzymes are involved in ROS generation and lignification. Systemic acquired resistance (SAR) and induced systemic resistance (ISR) are critical defense strategies mediated by ROS and hormones. Transcription factors such as nonexpresser of pathogenesis-related genes 1 (*NPR1*), TGACG-binding transcription factors 2 (TGA2), apetala 2 (AP2), and ethylene-responsive factor 1 (ERF1) regulate PR proteins like *PR1*, *PR2*, and *PR5*, as well as other defense-related genes like vegetative storage protein (VSP), lipoxygenase (LOX), and plant defensin 1.2 (PDF1.2). Red arrows indicate positive regulation, open blocks indicate negative regulation, dashed arrows represent direct interactions, and black arrows indicate a direct effect.

Basal resistance refers to a plant’s inherent ability to resist pathogen infection without prior exposure to the pathogen. This resistance is mediated by pre-existing physical and chemical barriers, along with a suite of constitutive defense mechanisms [18]. The involvement of ROS in basal resistance highlights their function as a primary line of defense against invading pathogens. ROS contributes to basal resistance through diverse mechanisms. ROS, particularly H_2_O_2_, can directly harm pathogens by disrupting their growth and development [19]. Aquaporins, specifically plasma membrane intrinsic proteins (PIPs), have been identified as key transporters of H_2_O_2_ across cell membranes. Also, a recent study uncovered that some aquaporins, such as AtPIP1;4 and AtPIP2;4, facilitate H_2_O_2_ across membranes and the translocation of externally applied H_2_O_2_ from the apoplast to the cytoplasm in *A*. *thaliana* during *Pseudomonas syringae* infection [20]. Additionally, studies on tomato plants (*Solanum lycopersicum* L.) have shown that the accumulation of ROS exerts antimicrobial effects on *Phytophthora infestans* [21]. Similarly, in wheat (*Triticum aestivum* L.) plants infected with powdery mildew, the rapid and localized generation of H_2_O_2_ at the sites of fungal penetration serves as an effective defense mechanism, restricting the pathogen’s ability to invade host tissues [22]. Although unstudied in plants, two key findings suggest ROS directly kill microbes in humans. First, pathogens lacking antioxidant defenses lose virulence, implying ROS-mediated damage. Second, oxidative harm—like lipid peroxidation, DNA breaks, and methionine oxidation—is detectable in microbes during respiratory bursts [23]. ROS trigger the biosynthesis of antimicrobial substances, such as phytoalexins, which further hinder pathogen proliferation. Additionally, the generation of ROS upon pathogen recognition initiates a signaling cascade that activates various defense responses, including hypersensitive reaction, the production of pathogenesis-related (PR) proteins, and the strengthening of cell walls (Figure 1) [22,24].

Structural barriers constitute a crucial element of basal resistance, as they physically obstruct pathogen invasion. Pathogen-induced ROS generation prompts rapid peroxidase-catalyzed cross-linking of cell wall proteins, reinforcing the cell wall and fortifying this physical barrier [24]. Furthermore, ROS can induce the deposition of lignin and suberin, thickening and strengthening the cell wall, particularly after pathogen infection [25]. Dirigent Protein 7 (DIR7) is a member of the dirigent protein family, which is involved in the biosynthesis of lignin and lignans. These proteins contribute to the strengthening of cell wall structure by facilitating lignin formation and are particularly activated in response to environmental stresses and pathogenic infections. In addition, DIR7 contributes to the maintenance of cell wall integrity by synthesizing defense-related compounds, such as lignans and lignin, which are crucial for cell wall reinforcement, and regulating cell wall metabolism through the modulation of lignin biosynthesis and deposition, especially under stress conditions like pathogen infection. This regulation enhances the structural integrity of the cell wall, providing a robust barrier against pathogens and supporting plant defense responses [26,27]. In peppers (*Capsicum annuum* L.), suppression of *CaDIR7* reduced plant defense mechanisms, leading to susceptibility to *Phytophthora capsici* and salt stress [28].

The GhUMC1 gene (encoding a blue-copper protein) in cotton (*Gossypium hirsutum* L.) is homologous to the *AtBCB* gene in *Arabidopsis* and plays a crucial role in regulating hydrogen peroxide (H_2_O_2_) levels, JA signaling, and lignin metabolism during pathogen infection stress [29,30]; silencing *GhUMC1* increases susceptibility to *Verticillium dahliae*, down-regulates genes in the JA and SA signaling pathways, decreases transcripts of lignin synthesis genes, and lowers lignin content [31]. Both DIR7 and GhUMC1 proteins contribute to maintaining cell wall integrity and ROS signaling. However, it remains unclear whether specific lignin molecules trigger a surge in ROS or if ROS drive enhanced lignin deposition. The polymerization of monolignols into lignin is facilitated by H_2_O_2_, which serves as a detoxification process for ROS [32]. Certain enzymes implicated in the monolignol biosynthesis pathway, including *p*-coumarate 3-hydroxylase, caffeoyl shikimate esterase, and cinnamoyl-CoA reductase, have been linked to this mechanism [33].

Additionally, ROS have been proposed to serve multiple roles, including acting as antimicrobial molecules that directly damage pathogen cells [23], cross-linkers in the plant cell wall that prevent pathogen invasion, and secondary messengers that activate further immune responses [1].

### 1.2. Antioxidant and Antimicrobial Compounds

Upon pathogen exposure, ROS activate the production and function of plant antimicrobial phytochemicals and increase their accumulation through various signaling pathways, including MAP kinase cascades, ST/JA signaling, and the SA pathway [33]. These compounds, such as flavonoids and other phenolic compounds like SA, act as signaling molecules to activate and/or increase the expression of downstream genes involved in phytoalexin biosynthesis. For instance, Yang et al. (2017) [34] observed that ET signaling in rice plants (*Oryza sativa* L.) upregulates the expression of the transcription factor (TF) OsEIL1 following invasion by the fungal pathogen *M. oryzae*. This TF directly binds to the promoters of *OsrbohA/B* (*Rboh* genes) and phytoalexin biosynthesis genes such as *OsOPR4* (encodes 12-oxophytodienoate reductase), linking ROS production to diterpenoid phytoalexin accumulation.

ROS alters the expression of TFs related to redox-sensitive or MAP kinase cascades, thereby increasing the activity of key enzymes of phytoalexin biosynthesis such as phenylalanine ammonia-lyase (PAL) and cytochrome P450 monooxygenases (Figure 1) [35,36]. On the other hand, excessive accumulation of ROS compounds leads to oxidation of cellular components. Therefore, plant antioxidant systems, comprising non-enzymatic scavengers (such as polyphenols, flavonoids, tannins, terpenoids, alkaloids, glutathione, and ascorbate) and enzymatic scavengers (such as APX, POD, CAT, and SOD), mitigate ROS levels in cells to prevent oxidative damage while preserving signaling pathways essential for induction of these antimicrobial phytochemicals and phytoalexins [11,16].

Interestingly, ROS dynamics in spatial and temporal are important in plant-pathogen interactions. Local oxidative bursts are often accompanied by the deposition of phytoalexins at the wound site, which creates a toxic microenvironment to control pathogen infection. In contrast, ROS waves play a systemic role in defense signaling, stimulating phytoalexin synthesis pathways and prompting responses in distal tissues [37]. These interplays highlight ROS as both a direct antimicrobial agent and a coordinator of phytochemical defenses. While moderate ROS levels are essential for signaling pathways that induce antimicrobial compound synthesis, excessive ROS accumulation can disrupt these pathways through multiple mechanisms. Excessive ROS can damage critical cellular components, including DNA, RNA, proteins, and lipids, which directly impact the metabolic machinery involved in phytoalexin biosynthesis. This oxidative damage can lead to enzyme deactivation, particularly those cytochrome P450 monooxygenases and other enzymes critical in phytoalexin production pathways [38]. Additionally, excessive ROS-induced redox imbalance can interfere with the transcriptional regulation of defense genes, potentially suppressing the expression of key enzymes in antimicrobial biosynthetic pathways.

### 1.3. ROS as a Signaling Molecule in Phytochemical Induction

Phytochemicals—a broad class of plant-derived secondary metabolites—include compounds such as phytoalexins. Phytochemicals include phenolic compounds [such as polyphenols (flavonoids and tannins)], terpenoids, flavonolignans, alkaloids, steroids, saponins, glycosides, lignins, and phenylpropanoid glycerols. Among these, phenolic and terpenoid compounds play essential molecular and biochemical roles in plants, including scavenging free radicals, facilitating signaling, regulating auxin transport, and enhancing plant defense mechanisms [39].

ROS serve as signals because they can modifying various proteins, including protein phosphatases, transcription factors, and protein kinases, regulating their activity [1]. Signaling may also involve the production of secondary metabolites, including phytoalexins and antioxidants, that are involved in plant defense and stress tolerance [40]. ROS, for instance, triggers the biosynthesis of one such component, lignin, a complex polymer that toughens cell wall preparation against an infectious agent via response to pathogens [1,41]. On the other hand, plant hormones, such as jasmonic acid and SA, can provoke ROS production, which may trigger the biosynthesis of defense-related phytochemicals [40,42]. Surprisingly, ROS participates in the biosynthesis of oxylipins, including jasmonates, important in plant defense and stress response [43]. Evidence from other systems shows OPDA (12-oxo-phytodienoic acid) can stabilize RBOH proteins, boosting ROS production and creating a defense-amplifying feedback loop. This crosstalk potentially explains ethylene-jasmonate convergence in upregulating diterpenoid pathways, though rice-specific validation remains needed. In addition, ROS-related pathways can stimulate the production of carotenoids and tocopherols, which are involved in ROS detoxification, and flavonols and anthocyanins are known for their ROS-scavenging properties [40].

ROS can directly influence the signal cascades by interacting with proteins or indirectly because of changes in the synthesis of phenolic compounds, which are highly important phytochemicals in plant defense mechanisms. Plants may synthesize these compounds under stress conditions as part of their defense mechanisms [40]. In other words, ROS could act as a prooxidant on such compounds, leading to an increased formation of ROS that contributes to a complex interaction essential for both plant defense and signaling pathways [1].

ROS can modulate the signaling cascades by interacting with proteins, which in turn can activate genes involved in the synthesis of flavonoids and anthocyanins. For example, in *A. thaliana*, experiments have shown that ROS accumulation triggers the expression of late biosynthetic and regulatory genes in the anthocyanin pathway. Mutants deficient in anthocyanins, such as *tt8-6* (a mutant of the *TT8* gene, encoding a bHLH transcription factor), show increased sensitivity to ROS-generating stresses. Conversely, the *pap1-D* mutant, which overexpresses the *PAP1* gene (encoding a MYB transcription factor), accumulates more anthocyanins and exhibits enhanced tolerance to these stresses, indicating a feedback regulation mechanism [44].

Redox-sensitive transcription factors activated by ROS can stimulate the transcription of genes involved in their synthesis [42]. This relationship functions as a feedback loop. When ROS levels increase, it signals for increased synthesis of these compounds, which then scavenge ROS and help regulate ROS levels, maintaining cellular homeostasis [40]. During pathogen infection, the initial burst of ROS serves as a signal to trigger defense mechanisms such as flavonoid and anthocyanin biosynthesis [1]. These then may scavenge ROS and contribute to the containment of both pathogen- and plant-originated damage [40]. These flavonoids can also be utilized as signaling molecules in the plant-microbe interaction process to influence the expression of other genes related to defense compounds’ biosynthesis pathways [44]. Therefore, phytochemicals exhibit pleiotropic effects; beyond their antimicrobial properties, they modulate ROS signaling to activate either survival mechanisms or pro-autophagic and pro-apoptotic pathways, depending on the oxidative stress-responsive capacity of the target cell. Pathogen attack can induce ROS signaling that influences the expression of genes responsible for flavonoid and anthocyanin biosynthesis [42]. It has been shown that ROS are involved in the activation of the MAPK cascade upon infection. Among the well-characterized MAPK cascades, AtMPK3/4/6 in Arabidopsis thaliana and OsMKK4-OsMPK3/6 in rice are key regulators of phytoalexin biosynthesis, ultimately controlling the expression of essential biosynthetic genes. Furthermore, ROS-mediated signaling is considered to target the transcription factors such as *AtWRKY33* and *OsTGAP1* further downstream of the MAPKs to control the phytoalexin biosynthesis [45].

### 1.4. Hypersensitive Response (HR)

Studying ROS in plant-pathogen interactions is crucial because ROS play a key role in plant defense mechanisms. A key early reaction of plants to pathogen invasion is the “oxidative burst”, characterized by a temporary and localized generation of ROS [2]. This burst is often associated with pathogen recognition and the hypersensitive response, a form of programmed cell death that limits pathogen spread [22]. The HR represents a localized programmed cell death (PCD) response that occurs at the pathogen infection site. It is a powerful defense mechanism that restricts pathogen spread and is often associated with resistance to biotrophic pathogens such as *Pseudoperonospora cubensis* [17,46]. The HR, driven by a ROS burst, is a dramatic example of how plants utilize ROS to actively combat pathogen invasion. ROS modulate various signaling pathways and cellular processes involved in programmed cell death [1]. This includes the activation of proteases, nucleases, and other enzymes involved in dismantling the cell. Maize (*Zea mays* L.) exhibits ROS production and a hypersensitive response upon encountering fungal pathogens like *Cochliobolus heterostrophus* [22,47]. Similarly, tobacco (*Nicotiana tabacum* L.) plants trigger ROS and the hypersensitive response in response to bacterial pathogens like *P. syringae* [48]. Tomato plants also display an oxidative burst and hypersensitive response when infected by the oomycete pathogen *Phytophthora infestans* [49]. Necrotrophic pathogens, such as *Alternaria solani*, can induce oxidative bursts in tomato leaves, leading to HR response and the development of fungal infection [16,50,51]. Interestingly, these defensive responses are not limited to just pathogens, as they have also been observed in response to beneficial microorganisms like rhizobia and mycorrhizal fungi [47]. The contrasting HR responses between biotrophic and necrotrophic pathogens are illustrated in Table 1.

### 1.5. Induced Resistance

ROS are instrumental in activating plant-induced resistance systems, such as SAR and ISR, during pathogen invasions (Figure 1) [16,52]. These processes enhance a plant’s capacity to withstand biotic stress through complex signaling pathways. SAR is a defense response induced after a localized pathogen infection, improving resistance across the entire plant.

When a pathogen triggers a localized oxidative burst, it can lead to the creation of “microbursts” of ROS in the leaf tissue. These microbursts are responsible for initiating micro-hypersensitive responses (micro-HRs), which occur before the development of SAR [53]. Hypersensitive responses linked to microbursts (micro-HRs) occurred before the onset of systemic acquired resistance. When the Rboh inhibitor was co-infiltrated with avirulent *P. syringae* in tomato (*S. lycopersicum* L.) leaves, it reduced the local oxidative burst and impeded the generation of systemic microbursts, thus hindering the development of SAR [22]. Conversely, the localized infiltration of H_2_O_2_ triggered systemic microbursts and activated SAR in primary leaves. This enhanced resistance in secondary leaves to subsequent infection by virulent strains of *P. syringae* [54]. This indicates that a signaling network mediated by ROS may be vital for improving plant resistance to infections. Consequently, changes in redox status or the buildup of ROS caused by pathogens can influence multiple facets of both local and systemic defense mechanisms [55].

ROS and SA are believed to play a key role in SAR [56]. Alterations in the oxidation state of the cell lead to the oligomerization of the NPR1 protein, a master regulator of SA-dependent gene expression, allowing it to translocate into the cell nucleus [57]. Upon arrival, NPR1 engages with TGACG-binding (TGA) transcription factors to activate the expression of defense-related genes [58]. Parallel to the activation of SAR, researchers observed bursts of ROS and localized cell death in seemingly uninfected tissues of the tomato plant [16,59]. These responses were prevented by inhibiting Rboh activity, which is responsible for ROS production.

Besides SA, JA, and ET are commonly associated with the activation of defense mechanisms in plants. The JA-dependent defense system, known as ISR, serves as a mechanism for plants to defend against necrotrophic pathogens and pests [60]. ROS accumulation can induce the expression of genes involved in the biosynthesis of JA. For instance, ROS can enhance the activity of lipoxygenase (LOX), which converts fatty acids into JA precursors, thereby increasing JA levels during pathogen stress [61]. Regulating ROS levels could amplify the JA-associated defense response of tomato plants when exposed to *Alternaria solani*-induced stress [16]. Also, extracellular self-DNA in plants has been demonstrated to induce the generation of ROS, which in turn stimulates JA signaling pathways. This activation prompts the expression of JA-responsive genes that are essential for plant immunity against pathogens such as *Botrytis cinerea* and *P. syringae* [62]. However, the interaction between ROS and other signaling molecules, such as SA and ET, gives rise to a complex regulatory network that shapes plant responses to pathogenic threats [63]. For instance, during infection by the necrotrophic pathogen *Botrytis cinerea* in *Arabidopsis*, ROS accumulation in the apoplast, driven by RBOHs like RBOHD, interacts with ET signaling to enhance necrosis and disease progression, as ET amplifies ROS-mediated cell death [50]. In contrast, when *Arabidopsis* faces the biotrophic pathogen *P. syringae*, ROS production collaborates with SA to trigger SAR, where SA-dependent pathways amplify ROS signals via feedback loops involving *NPR1*, a key regulator of SA signaling, to bolster defense gene expression [64]. The interplay among these signaling molecules can either potentiate or suppress the JA-dependent defense mechanism.

SAR is primarily mediated by the SA. When plants detect pathogens, they begin producing ROS, which then triggers the accumulation of SA. This accumulation of SA then activates the transcription factor *NPR1* and other regulatory proteins, leading to the upregulation of defense-related genes like *PR1* and *PAL*. The proteins produced by these genes are crucial in enhancing the plant’s defenses against a wide range of pathogens. However, ISR is often associated with beneficial microorganisms and involves signaling pathways mediated by the JA. ROS also play a role in this process by priming the plant’s immune system. Key regulatory genes, such as *JAR1* and *EIN2*, are involved in this pathway, ultimately leading to the upregulation of defense-related genes like *PDF1*.2 and *lipoxygenases*, which prepare the plant for potential future pathogen encounters (Table 2).

### 1.6. Exploitation of ROS by Pathogens in Infected Plants

The interaction between plants and pathogens is complex, with ROS playing a pivotal role. During early infection, plants often initiate an “oxidative burst”, rapidly accumulating ROS like superoxide and hydrogen peroxide. However, pathogens have developed strategies to counter this defense by producing ROS-scavenging enzymes or down-regulating the plant’s ROS-producing systems.

When plant cells detect microbe-associated molecular patterns (MAMPs), they initiate an oxidative burst that results in the generation of ROS. Avirulent pathogens, recognized by the plant’s immune system, induce biphasic ROS accumulation. The initial phase involves a brief, low-intensity reaction, while the subsequent phase features a significantly greater and prolonged buildup of ROS [63]. Virulent pathogens, which bypass host detection, only induce the initial, temporary phase of ROS production. This suggests a role for ROS in initiating plant defenses [65].

However, some bacterial pathogens employ various strategies to suppress ROS production in plants. For instance, the bacterial effector AvrPtoB from *Pseudomonas* can suppress ROS production triggered by PAMP recognition [66]. Another example is the *Pseudomonas* effector HopA1, which dephosphorylates and inactivates *Arabidopsis MAPK3* and *MPK6* at the protein level, essential components of the ROS signaling pathway [67]. Pathogens employ strategies to manipulate the plant’s ROS signaling pathways. The bacterial pathogen *Pseudomonas* produces coronatine, a virulence factor that mimics the action of the JA. This compound then suppresses the plant’s SA-mediated defense responses, including those involving ROS [68]. Also, some fungal pathogens can interfere with plant ROS production by secreting effector proteins. The fungus *Claviceps purpurea* uses the transcription factor CPTF1 to regulate the expression of an H_2_O_2_-inducible *CAT* gene (cpcat1), which helps the pathogen cope with the rye (*Secale cereale* L.) oxidative burst during infection [69]. Necrotrophic pathogens benefit from host cell death. They can induce ROS production in the infected tissue to promote cell death, facilitating their infection process [51]. For instance, the necrotrophic fungus *Botrytis* induces ROS production through Rboh activity, leading to cell death and enhanced susceptibility [70].

### 1.7. Interplay of ROS with Phytohormones and Phytochemicals: Pairwise and Multidirectional Interactions

ROS and phytohormones (SA, JA, ET) engage in bidirectional regulation: Hormones can stimulate ROS generation through NADPH oxidases, while ROS waves activate hormone signaling—a pivotal mechanism in SAR. Figure 1 illustrates the dynamic interactions among ROS, phytohormones, and phytochemicals that orchestrate plant defense responses during pathogen challenges. Although SA is a prime regulator of ROS, the coordinated mechanisms are still in their infancy [71]. A key target of SA in triggering ROS signaling is the NADPH oxidase RBOH [72]. Myers et al. (2023) found that JA and SA have opposing effects on the ROS wave—JA suppresses it during high light or wounding stress; while SA enhances it [73]. Additionally, ET regulates the ROS wave in response to wounding but not high-light stress.

The most prevalent and varied phytoalexins are low-molecular-weight phytochemicals, notably polyphenols and terpenoids. A recent review comprehensively outlines the diverse structures and health-promoting properties of these bioactive compounds [74]. The three-way interplay between phytochemicals, phytohormones, and ROS is highly complex and challenging to fully elucidate. Preliminary experiments reveal that phytohormone-mediated flavonoid metabolites boost plant growth and anti-herbivore defense via ROS modulation [75]. Research suggests ET signaling contributes to disease resistance by triggering ROS and phytoalexin production in rice infected with *Magnaporthe oryzae* [34]. Given the lack of studies on ROS-phytochemical interactions in plants, we draw parallels from human cancer research, where ROS-mediated cell death mechanisms combat malignancies, a concept that could inspire future phytochemical studies in plant systems. Polyphenols, flavonoids, and stilbenes boost ROS production, selectively triggering apoptosis and autophagy in cancer cells in humans [76]. Emerging evidence suggests that microRNA regulation may amplify the anticancer activity of polyphenols via ROS-dependent mechanisms, potentially in an additive or synergistic fashion [77].

## 2. Perspective and Future Directions of ROS in Crop Protection

Genetic modifications can be utilized to modulate ROS levels in plants, thereby enhancing their defense against pathogens. Overexpression of genes associated with ROS generation, such as *Rboh* genes encoding Rboh enzymes, can result in elevated ROS accumulation and improved resistance to select pathogens [78]. In contrast, repressing genes that encode enzymes inhibiting ROS production can also enhance disease resistance against specific pathogens. For instance, *A. thaliana* CAT mutants (*cat2* and *cat3*) exhibit increased cell death in response to *P. syringae* pv. tomato DC3000 (Pst) expressing AvrRpm1, with reduced bacterial growth compared to wild-type plants, indicating enhanced resistance [79]. Also, a study on wheat (*T. aestivum* L.) revealed that disrupting the *TaWRKY19* gene, a gene encoding an enzyme involved in ROS production, led to elevated ROS levels and enhanced resistance against stripe rust (*Puccinia striiformis*) [80]. Precisely engineering ROS signaling pathways through genetic modification holds the potential for enhancing plant defense mechanisms. By manipulating genes that encode proteins involved in ROS sensing, signal transduction, and downstream effects, researchers can optimize ROS-mediated resistance. For instance, modulating the expression of *OsRac1* in rice, which encodes a protein that interacts with and modulates the activity of OsRBOHB, can influence ROS production. Specifically, overexpression of *OsRac* can enhance OsRBOHB activity, leading to increased ROS generation, while its down-regulation may reduce ROS levels by inhibiting OsRBOHB activity [81], thereby impacting disease resistance.

While increasing ROS levels can be beneficial for defense, it is crucial to maintain a balance to avoid detrimental effects on plant health. Genetic engineering can be used to enhance the antioxidant capacity of plants, ensuring that ROS levels remain within a safe range [16]. This can be achieved by overexpressing genes encoding antioxidant enzymes, such as SOD, APX, and CAT. However, critical frontiers must be addressed to translate ROS manipulation into robust agricultural solutions. Current genetic tools lack precision in timing and localizing ROS signals. Optogenetic systems using CRY2/CIB1 photoreceptors could enable light-activated ROS generation specifically at fungal infection sites in wheat leaves. Preliminary models suggest this approach may reduce off-target oxidative damage by 80% compared to constitutive overexpression [82]. ROS interacts with 23+ hormonal pathways, creating unpredictable trade-offs. In salt-stressed barley, engineered *HvRBOHF* lines showed enhanced *Blumeria graminis* resistance but exacerbated ABA-mediated stomatal closure, reducing CO_2_ assimilation by 25% [83]. Systems biology frameworks integrating multi-omic datasets are urgently needed to predict these interactions. Most ROS-modified crops are tested against single pathogens in controlled environments. When deployed in flood-prone Bangladeshi rice paddies, *OsRac1*-silenced lines maintained 90% blast resistance in lab trials but showed only 40–50% efficacy under concurrent salinity and arsenic stress [82,84]. Next-generation designs must incorporate stress-resilient redox buffers that function across abiotic-biotic stress combinations.

## 3. Conclusions

Under certain conditions, ROS play dual roles, acting as both beneficial and harmful factors in plant health. Excessive ROS may play a critical role in the pathogenesis of the host plant while also serving to inhibit pathogen growth. Given the pleiotropic roles of ROS in plant-pathogen interactions, future plant disease control strategies should focus on modulating the redox state in infected tissues. The interplay between ROS and hormones (SA, JA, and ET) in plant-pathogen interactions modulates defense signaling, orchestrating immune responses such as PCD, PR gene activation, and systemic resistance. Phytochemicals also hold promise for developing crop cultivars with enhanced disease resistance, given their multiple roles in priming of defense responses, direct pathogen inhibition, and reinforcement of physical barriers. Nevertheless, the interplay among ROS, phytohormones, and phytochemicals is complex and not yet fully resolved. Nevertheless, the interplay among ROS, phytohormones, and phytochemicals remains highly complex. Future studies combining advanced omics and real-time redox imaging could yield deeper mechanistic insights.

## Figures and Tables

**Table 1:** Hypersensitive response dynamics in biotroph vs. necrotroph interactions.

Feature	Biotrophs	Necrotrophs
Pathogen strategy	Sustain host viability	Kill the host cell/tissue
Plant hormone	SA-dominated	JA/ET-dominated
ROS role	Direct antimicrobial action + PCD	Secondary ROS exacerbates + PCD
HR occurrence	Early, localized	Limited (risk of pathogen benefit)
Outcome	Effective containment	Often detrimental to the plant

PCD, Programmed cell death; ROS, Reactive oxygen species; HR, Hypersensitive response; SA, Salicylic acid; JA, Jasmonic acid; ET, Ethylene.

**Table 2:** Simplified model of ROS-induced systemic acquired resistance (SAR) and induced systemic resistance (ISR) pathways.

Component	Systemic Acquired Resistance (SAR)	Induced Systemic Resistance (ISR)	Reference
Pathway	SA pathway	JA and ET pathways	[19]
Key chemicals	SA, ROS	JA, ET, ROS	[59]
Transcription factors	NPR1, TGA1/TGA4	EIN3, JAZ proteins, MYC2	[16]
Induced genes	*PR1*, *PR2*, *PAL*, *ICS1*	*JAR1*, *EIN2*, *FAD*, *PDF1.2*	[19]
Proteins	PR proteins (e.g., PR1), ICS	LOX, JAZ protein	[16]

The abbreviations represent the following: NPR1, Non-expression of pathogenesis-related genes 1; TGA1/TGA4, TGACG-Binding Factor 1/TGACG-Binding Factor 4; PR1, Pathogenesis-Related protein 1; PR2, Pathogenesis-related protein 2; PAL, Phenylalanine ammonia-lyase; ICS1, Isochorismate synthase 1; PR proteins, Pathogenesis-related proteins; EIN3 protein, Ethylene insensitive 3 protein; JAZ protein, Jasmonate ZIM-Domain protein; MYC2, MYC Transcription Factor 2; JAR1, Jasmonic acid-amido synthetase1, EIN2, Ethylene insensitive 2; FAD, Flavin adenine dinucleotide; and PDF1.2, Plant Defensin 1.2.

## Data Availability

Data are contained within the article.
